# Weight trends among adults with diabetes or hypertension during the COVID-19 pandemic: an observational study using OpenSAFELY

**DOI:** 10.3399/BJGP.2023.0492

**Published:** 2024-10-01

**Authors:** Miriam Samuel, Robin Y Park, Sophie V Eastwood, Fabiola Eto, Caroline E Morton, Daniel Stow, Sebastian Bacon, Ben Goldacre, Amir Mehrkar, Jessica Morley, Iain Dillingham, Peter Inglesby, William J Hulme, Kamlesh Khunti, Rohini Mathur, Jonathan Valabhji, Brian MacKenna, Sarah Finer, the OpenSAFELY Collaborative

**Affiliations:** Wolfson Institute of Population Health, Queen Mary University of London, London.; Bennett Institute for Applied Data Science, Nuffield Department of Primary Care Health Sciences, University of Oxford, Oxford.; MRC Unit for Lifelong Health and Ageing, University College London, London.; Wolfson Institute of Population Health, Queen Mary University of London, London.; Wolfson Institute of Population Health, Queen Mary University of London, London.; Wolfson Institute of Population Health, Queen Mary University of London, London.; Bennett Institute for Applied Data Science, Nuffield Department of Primary Care Health Sciences, University of Oxford, Oxford.; Bennett Institute for Applied Data Science, Nuffield Department of Primary Care Health Sciences, University of Oxford, Oxford.; Bennett Institute for Applied Data Science, Nuffield Department of Primary Care Health Sciences, University of Oxford, Oxford.; Bennett Institute for Applied Data Science, Nuffield Department of Primary Care Health Sciences, University of Oxford, Oxford.; Bennett Institute for Applied Data Science, Nuffield Department of Primary Care Health Sciences, University of Oxford, Oxford.; Bennett Institute for Applied Data Science, Nuffield Department of Primary Care Health Sciences, University of Oxford, Oxford.; Bennett Institute for Applied Data Science, Nuffield Department of Primary Care Health Sciences, University of Oxford, Oxford.; Leicester Diabetes Centre, Leicester General Hospital and Diabetes Research Centre, College of Medicine, Biological Sciences and Psychology, University of Leicester, Leicester.; Wolfson Institute of Population Health, Queen Mary University of London, London.; Division of Metabolism, Digestion and Reproduction, Imperial College London, London.; Bennett Institute for Applied Data Science, Nuffield Department of Primary Care Health Sciences, University of Oxford, Oxford.; Wolfson Institute of Population Health, Queen Mary University of London, London.

**Keywords:** body mass index, COVID-19, health inequalities, hypertension, primary health care, type 2 diabetes mellitus

## Abstract

**Background:**

COVID-19 pandemic restrictions may have influenced behaviours related to weight.

**Aim:**

To describe patterns of weight change among adults living in England with type 2 diabetes (T2D) and/or hypertension during the pandemic.

**Design and setting:**

An observational cohort study using the routinely collected health data of approximately 40% of adults living in England, accessed through the OpenSAFELY service inside TPP.

**Method:**

Clinical and sociodemographic characteristics associated with rapid weight gain (>0.5 kg/m^2^/year) were investigated using multivariable logistic regression.

**Results:**

Data were extracted on adults with T2D (*n* = 1 231 455, 43.9% female, and 76.0% White British) or hypertension (*n* = 3 558 405, 49.7% female, and 84.3% White British). Adults with T2D lost weight overall (median δ = −0.1 kg/m^2^/year [interquartile range {IQR} −0.7–0.4]). However, rapid weight gain was common (20.7%) and associated with the following: sex (male versus female: adjusted odds ratio [aOR] 0.78 [95% confidence interval {CI} = 0.77 to 0.79]); age (older age reduced odds, for example, aged 60–69 years versus 18–29 years: aOR 0.66 [95% CI = 0.61 to 0.71]); deprivation (least deprived Index of Multiple Deprivation [IMD] quintile versus most deprived IMD quintile: aOR 0.87 [95% CI = 0.85 to 0.89]); White ethnicity (Black versus White: aOR 0.95 [95% CI = 0.92 to 0.98]); mental health conditions (for example, depression: aOR 1.13 [95% CI = 1.12 to 1.15]); and diabetes treatment (non-insulin treatment versus no pharmacological treatment: aOR 0.68 [95% CI = 0.67 to 0.69]). Adults with hypertension maintained stable weight overall (median δ = 0.0 kg/m^2^/year [IQR −0.6–0.5]); however, rapid weight gain was common (24.7%) and associated with similar characteristics as in T2D.

**Conclusion:**

Among adults living in England with T2D and/or hypertension, rapid pandemic weight gain was more common among females, younger adults, those living in more deprived areas, and those with mental health conditions.

## Introduction

Restrictions imposed to reduce COVID-19 transmission resulted in profound societal changes that may have influenced weight-related health behaviours.^1^^–^4 This is of particular relevance among adults with type 2 diabetes (T2D) and/or hypertension, conditions that are more common among adults with obesity, and interact with obesity to increase the risk of non-communicable disease^5^ and serious disease with COVID-19 infection.^6^

In addition to absolute weight, rate of weight gain is an independent risk factor for cardiovascular disease^7^ and predicts risk of progression to less healthy body mass index (BMI) categories.^8^ The authors of the present study previously reported health inequalities in patterns of pandemic weight gain among adults living in England, with females, those living in greater deprivation, and those with mental health conditions at greatest risk of rapid weight gain.^9^ Importantly, it was found that the risk of rapid weight gain was decreased with T2D but increased with hypertension.^9^ However, whether these trends apply in all sociodemographic groups of adults living with these conditions is unclear. Understanding which subgroups of adults living with T2D and/or hypertension were at greatest risk of unhealthy patterns of weight gain during the pandemic will support GPs’ target discussions and interventions on weight and health-related behaviours.

Routinely collected healthcare records were used to address the following questions among adults with T2D and/or hypertension in England during the COVID-19 pandemic:

1. What was the median BMI, and proportion obese, at a population level and within clinical and sociodemographic substrata?
2. What were individual-level patterns of weight change during the pandemic? And
3. Which sociodemographic and clinical characteristics were associated with an increased individual-level odds of rapid weight gain (>0.5 kg/m^2^/year) during the pandemic?

**Table 1:** How this fits in

The unequal impact of the COVID-19 pandemic on population health has been described. This study used English primary care records to investigate weight trends among adults living with hypertension and/or type 2 diabetes (T2D) during the pandemic. It observed that on average adults with hypertension maintained a stable weight during the pandemic, while those with T2D lost weight. However, underlying these trends, rapid weight gain was common (>0.5 kg/m^2^/year), with female sex, younger age, greater deprivation, and comorbid mental health conditions associated with an increased risk of rapid weight gain in both populations.

## Method

### Data Source

Primary care health records, managed by the GP software provider TPP, were linked, stored, and analysed securely within the NHS England COVID-19 OpenSAFELY service inside TPP (OpenSAFELY-TPP), containing pseudonymised data on approximately 40% of the English population, including sociodemographic data, coded diagnoses, medications, and physiological parameters. No free-text data were included. Detailed pseudonymised patient data are potentially re-identifiable and therefore not shared. An information governance statement is included (see Supplementary Information S1).

### Data management

Data management was done with Python (version 3.8) and SQL, and analysis was performed using R (version 4.0). Prevalence counts are rounded to the nearest five to reduce risk of disclosure. Numbers given for rounded components in individual strata of analyses may not add up to the rounded sum. All codes for data management and analysis, as well as codelists, are shared openly for review and re-used under MIT open licence, which is available at: https://github.com/opensafely/BMI-and-Metabolic-Markers.

### Study population

Data were extracted on all male and female adults aged 18–90 years, who were registered with a primary care practice, using TPP electronic health record (EHR) software for at least 1 year before 1 March 2022. Those who had a coded diagnosis of either T2D and/or hypertension in their EHRs were identified.

### Study outcomes: BMI

BMI (weight in kilograms [kg] divided by height in metres squared [m^2^]) is recorded in primary care records during routine health checks, disease monitoring, and when clinically indicated. BMI data were extracted between 1 March 2015 and 1 March 2022. BMI (kg/m^2^) values were obtained either by computing recorded weight and height measurements or, where they were not available, from recorded BMI values present. Weight and BMI measures from before an individual reached 18 years of age were ignored. Height measurements recorded on individuals aged >18 years before 1 March 2015 were included. Monthly BMI values were extracted per individual and, where multiple values per calendar month were present, the most recent value was taken. Extreme values (BMI <15 kg/m^2^ and BMI >65 kg/m^2^) were omitted from analyses to censor erroneous results and exclude extremely overweight and underweight individuals, whose patterns of weight change may not represent general population trends. It was found that <0.03% of adults with a BMI value recorded were subsequently excluded from analyses in any given year owing to omission of extreme values.

### Cross-sectional population-level BMI estimates

A cross-sectional approach was used to estimate the population-level median BMI and prevalence of obesity, to give context to the trajectory analysis. BMI values were first categorised by the year in which they were recorded (for example, March 2021–March 2022) and individuals were assigned a yearly BMI where data were available. Where multiple values were available in a given year for a single individual, the median was used as the yearly BMI. Owing to the reduction in BMI recording during the pandemic, population-level descriptive BMI statistics were estimated using the most recent yearly BMI recorded in the 5 years preceding March 2022. Individuals were classified as being obese if they had a BMI ≥30 kg/m^2^ (≥27.5 kg/m^2^ in Black, Asian, or ethnic minority individuals).^10^

### Individual-level BMI trajectories during the pandemic

**Figure 1.**
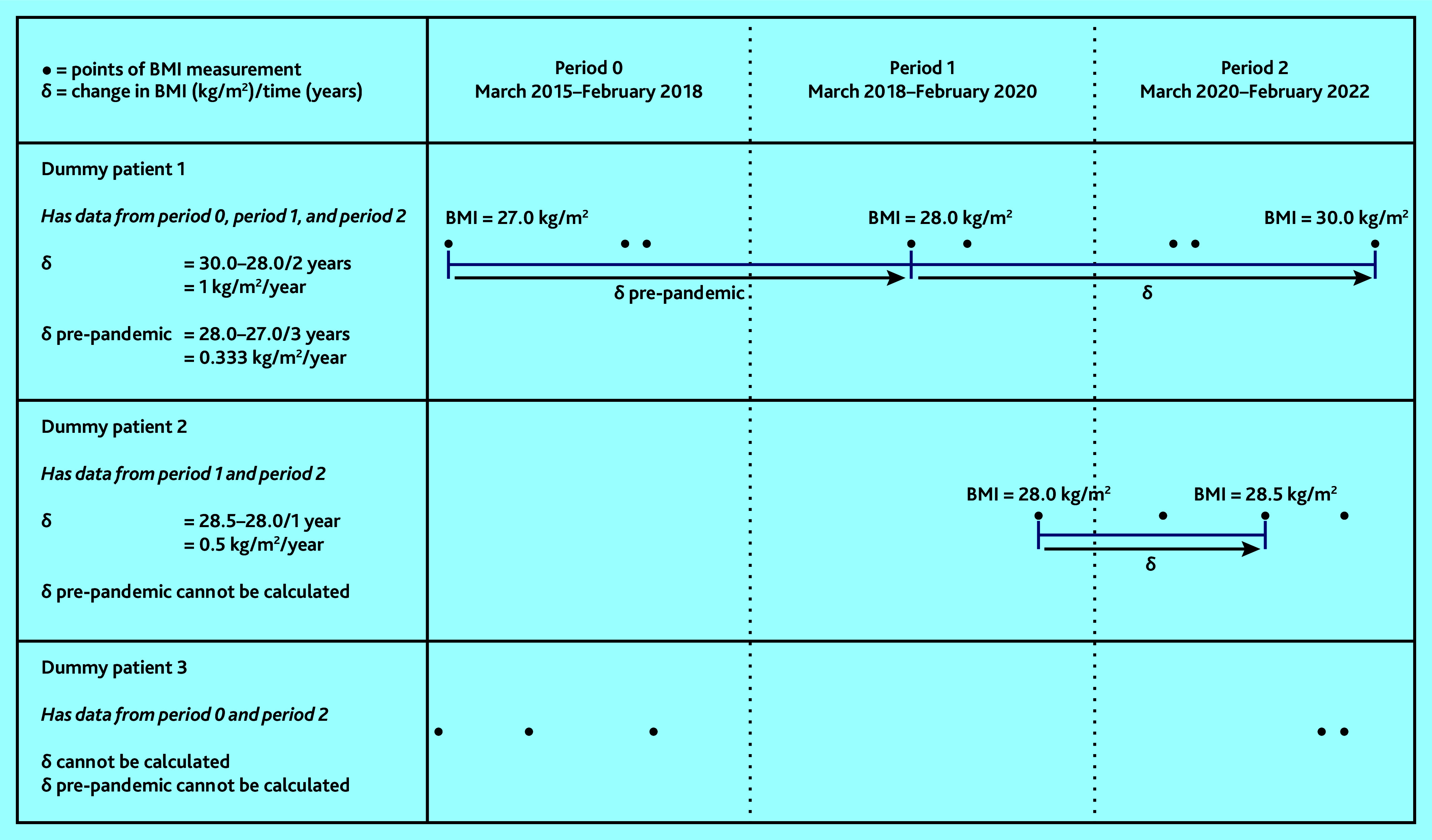
Calculation of rate of BMI change per year (δ) in kg/m^2^/year. BMI = body mass index.

Individual-level BMI trajectories were estimated as rates of BMI change per year (δ) in kg/m^2^/year. To calculate δ, recorded BMI values were categorised into the following time windows: period 1 (March 2018–February 2020) and period 2 (March 2020–February 2022); then, for individuals with BMI data available, one BMI value recorded in each of these periods was randomly selected. Among adults with a BMI value recorded in each of these periods, individual-level rates of weight change (δ) between period 1 and period 2 were then calculated, assuming a linear trend (Figure 1 and Supplementary Information S2).^8^^,^^9^ Individuals who were underweight (BMI <18.5 kg/m^2^) in period 1 and those with known cancer were excluded from these analyses as their patterns of weight change were likely to differ from the general population. The most extreme 0.05% of values (a positive or negative change of >6 kg/m^2^/year) were censored to reduce the impact of erroneous results. To further contextualise these results, the rate of weight gain in the pre-pandemic period was calculated using similar methods (see Figure 1 and Supplementary Information S2).

Using individual-level BMI trajectories, adults who gained >0.5 kg/m^2^/year were subsequently categorised as experiencing ‘rapid weight gain’. This rate is independently associated with cardiovascular morbidity.^7^

### Covariates

Covariates included age, sex, ethnicity (based on the 2001 UK Census definitions),^11^ deprivation (using patient postcode-derived Index of Multiple Deprivation [IMD]),^12^ and the presence of 14 long-term conditions ever recorded (Supplementary Information S3). Adults with T2D were further classified based on their prescribed diabetes medication regimen into the following three groups: insulin-based regimens (insulin); regimens that did not include insulin (non-insulin); and those who were not prescribed pharmacological treatment (Supplementary Information S3).

### Statistical models

Complete-case analysis was used, which only included individuals with all baseline covariate data. Complete data were available for age and sex, as these were part of the study inclusion criteria, and long-term conditions (such as depression), which were identified based on the presence or absence of specified clinical codes. Therefore, ethnicity and IMD were the only variables with missing data. The missing at random assumption (required for imputation) was unlikely to hold; for example, presence of recorded ethnicity is likely to be related to the ethnicity itself. However, the complete case assumption of independence between missingness and outcomes (conditional on the covariates) was more plausible.^13^

The baseline characteristics of the populations with T2D and/or hypertension was compared with and without the outcome (rapid pandemic weight gain). Further comparison of baseline characteristics and outcomes was made between the complete case sample and entire population, including those with missing ethnicity and deprivation data.

Logistic regression was used to explore associations between the sociodemographic and clinical covariates and the odds of rapid weight gain among adults with hypertension or T2D. Models were adjusted separately for age, sex, IMD, and ethnicity, and then in multivariable models adjusted for age and sex; age, sex, and IMD; age, sex, and ethnicity; and age, sex, IMD, and ethnicity.

### Subgroup analyses

It was investigated whether estimated associations between the covariates and extreme acceleration in rate of weight gain persisted in populations stratified by age group (18–39 years, 40–59 years, and 60–79 years), sex, and ethnicity (Black and South Asian).

### Patient and public involvement

OpenSAFELY has a publicly available website through which patients or members of the public were invited to contact the authors about this study or the broader OpenSAFELY project.

## Results

**Figure 2.**
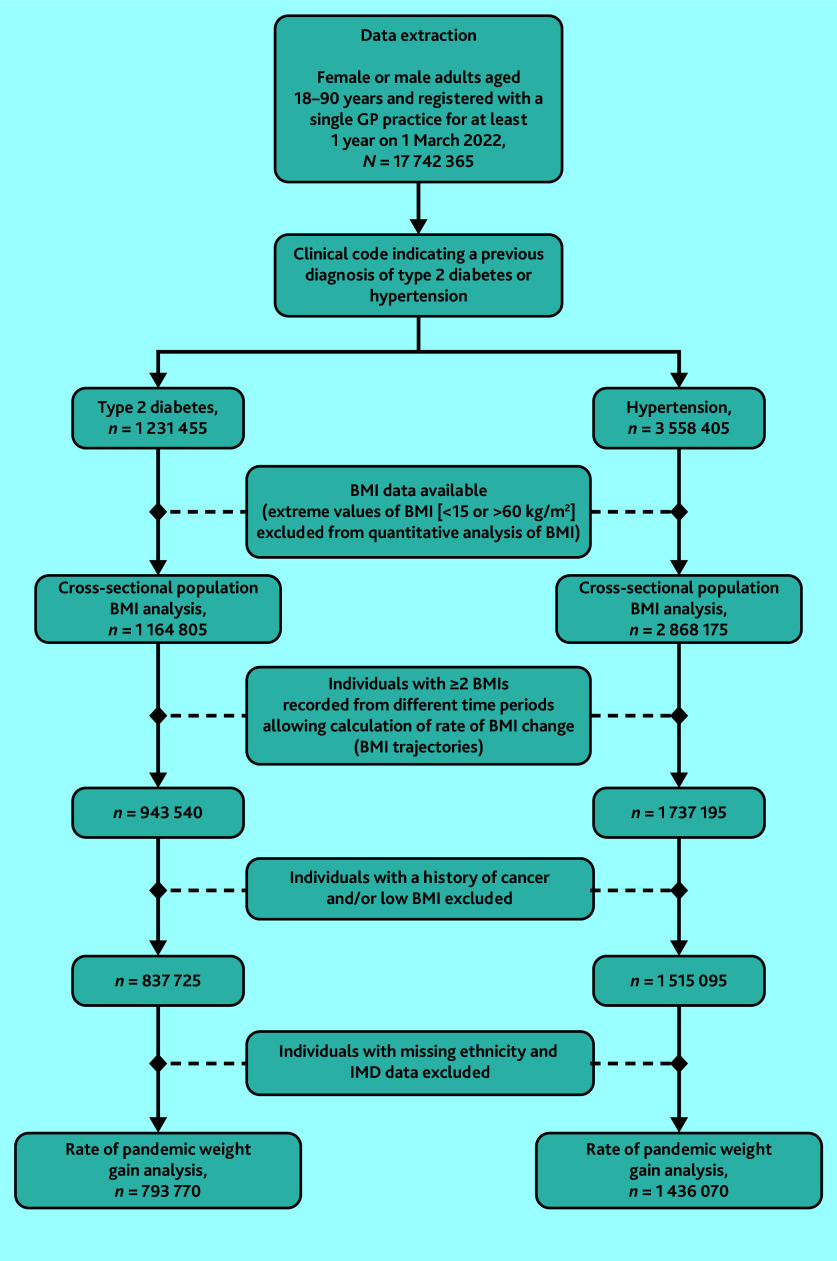
Selection of the study population. BMI = body mass index. IMD = Index of Multiple Deprivation.

Data were extracted on 1 231 455 adults with T2D (43.9% [*n* = 540 925] female and 76.0% [*n* = 902 010/1 187 125] White British; see Supplementary Table S1) and 3 558 405 adults with hypertension (49.7% [*n* = 1 767 845] female and 84.3% [*n* = 2 858 145] White British; see Supplementary Table S2), of whom 792 630 adults had both hypertension and T2D and were included in both arms of the analysis (Figure 2 and Supplementary Table S1). Among adults with T2D, 64.4% (*n* = 792 630) had comorbid hypertension, while 25.7% (*n* = 316 555) had comorbid depression. Among adults with hypertension, 22.3% (*n* = 792 630) had comorbid T2D, while 23.2% (*n* = 825 640) had comorbid depression. In the total study population, few adults with T2D had missing data for ethnicity (3.6%, *n* = 44 330) or IMD (2.0%, *n* = 24 360). Missing data for ethnicity (4.7%, *n* = 167 250) and IMD (1.9%, *n* = 68 495) were also uncommon among adults with hypertension (Supplementary Tables S1 and S2).

### Cross-sectional population BMI estimates

**Figure 3.**
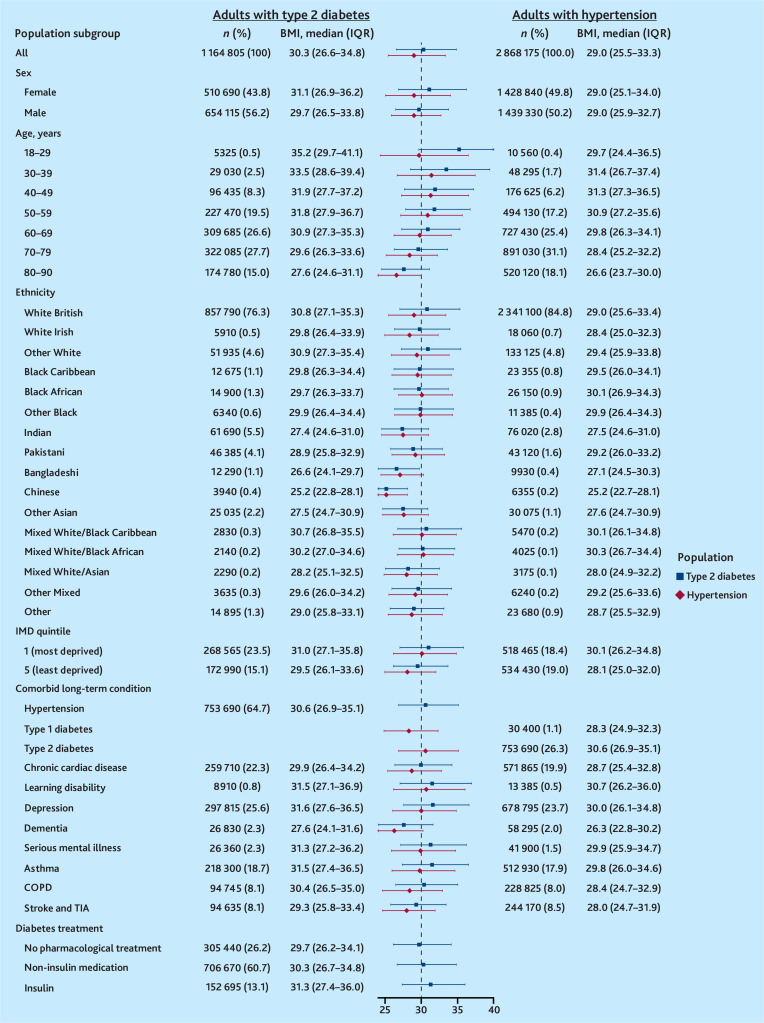
Population-level BMI estimates among people with type 2 diabetes and/or hypertension. BMI = body mass index. COPD = chronic obstructive pulmonary disease. IMD = Index of Multiple Deprivation. IQR = interquartile range. TIA = transient ischaemic attack.

Among adults with T2D, more than half were obese (55.5%, *n* = 646 805), median BMI 30.3 kg/m^2^ [interquartile range {IQR} 26.6–34.8]). BMI was higher among: younger adults (aged 18–29 years: 35.2 kg/m^2^ [IQR 29.7–41.1]); females (31.1 kg/m^2^ [IQR 26.9–36.2]); those in the most deprived IMD quintile (31.0 kg/m^2^ [IQR 27.1–35.8]); those with mental health conditions including depression (31.6 kg/m^2^ [IQR 27.6–36.5]); and those taking insulin (31.3 kg/m^2^ [IQR 27.4–36.0]). White adults living with T2D had the highest BMI (White British: 30.8 kg/m^2^ [IQR 27.1–35.3]), followed by those with a mixed White/Black Caribbean ethnicity (30.7 kg/m^2^ [IQR 26.8–35.5]) (see Figure 3 and Supplementary Table S3).

Among adults with hypertension, just under half were obese (45.2%, *n* = 1 297 010). BMI was higher among those living in the most deprived IMD quintile (30.1 kg/m^2^ [IQR 26.2–34.8]), and those living with comorbidities including T2D (30.6 kg/m^2^ [IQR 26.9–35.1]) and mental health conditions (for example, depression: 30.0 kg/m^2^ [IQR 26.1–34.8]). There was no difference in BMI between males and females with hypertension (Figure 3 and Supplementary Table S3).

### Individual-level BMI trajectories (δ) during the pandemic

**Figure 4.**
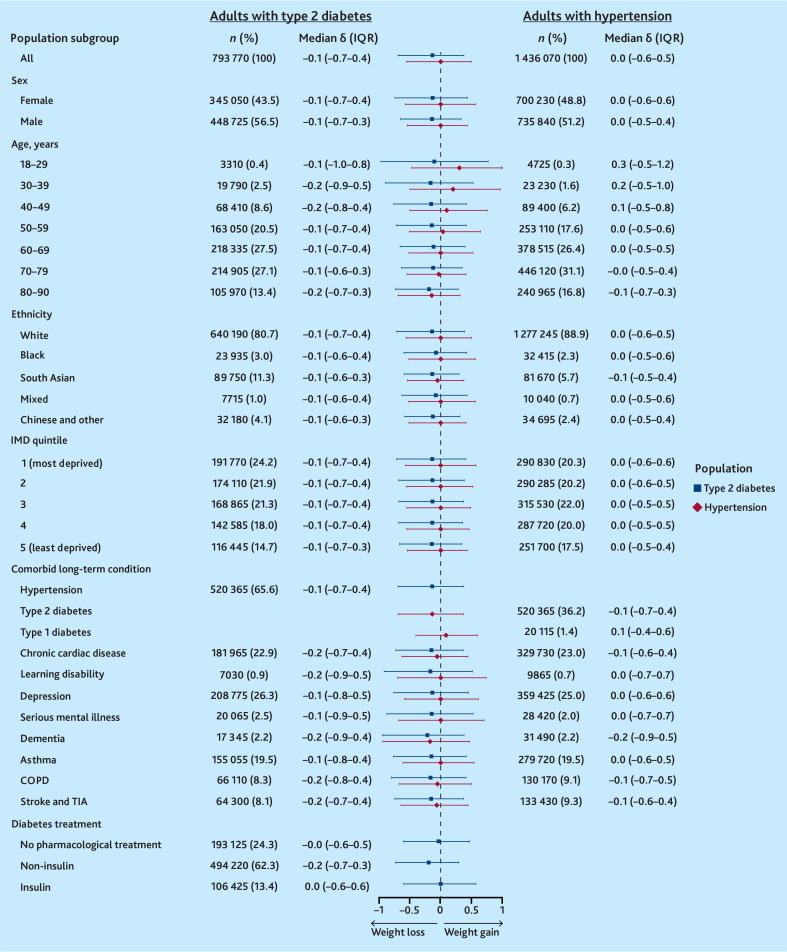
Average rate of weight gain (δ) during the COVID-19 pandemic among adults with diabetes and/or hypertension. BMI = body mass index. COPD = chronic obstructive pulmonary disease. IMD = Index of Multiple Deprivation. IQR = interquartile range. TIA = transient ischaemic attack.

During the pandemic, adults with T2D lost weight overall but there was a wide distribution of individual-level weight trajectories, with the upper quartile of adults gaining at least 0.4 kg/m^2^/year (median δ = −0.1 kg/m^2^/year [IQR −0.7–0.4]) (Figure 4). Only adults taking insulin and those receiving no pharmacological treatment for diabetes maintained a stable weight on average. In comparison, the distribution of individual-level BMI trajectories was narrower before the onset of the pandemic, although the median rate of weight change was the same (median δ pre-pandemic = −0.1 kg/m^2^/year [IQR −0.6–0.3]) (Supplementary Figure S1).

During the pandemic, adults with hypertension maintained a stable weight overall but there was also a wide distribution of individual-level weight trajectories, with the upper quartile of adults gaining at least 0.5 kg/m^2^/year (median δ = −0.0 kg/m^2^/year [IQR −0.6–0.5]) (Figure 4). Among adults with hypertension, those aged <50 years and those with comorbid type 1 diabetes gained weight, while those with comorbid T2D lost weight overall. As seen among adults with T2D, there was a narrower distribution of BMI trajectories before the onset of the pandemic, but overall adults with hypertension maintained a stable weight during this time (median δ pre-pandemic = 0.0 kg/m^2^/year [IQR −0.5–0.4]) (Supplementary Figure S1).

### Rapid weight gain (δ >0.5 kg/m^2^/year) during the pandemic

**Figure 5.**
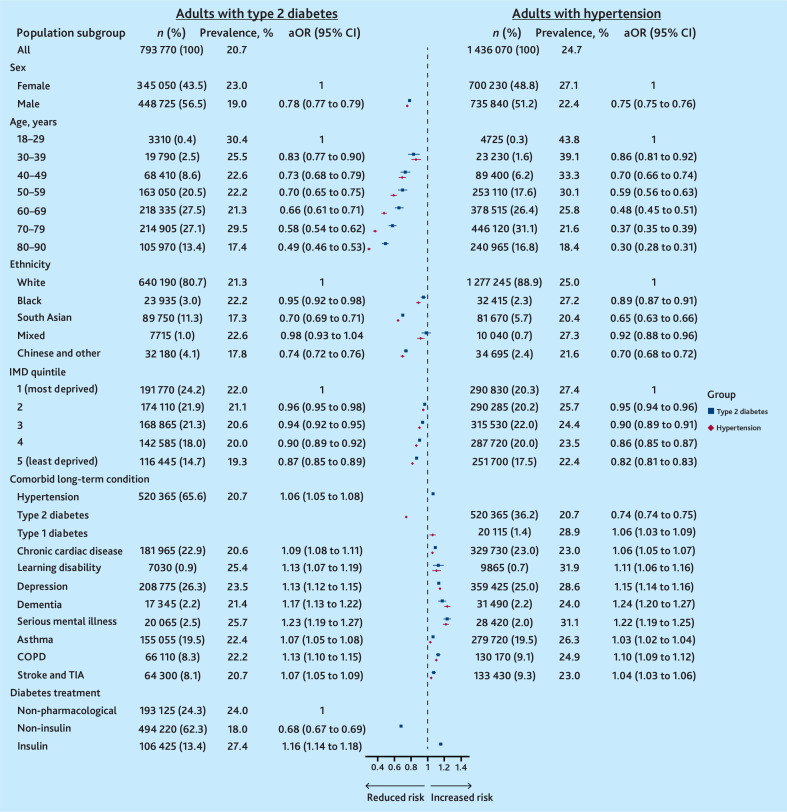
Prevalence and adjusted odds of rapid weight gain (>0.5 kg/m^2^/year) among adults living in England during the COVID-19 pandemic. aOR = adjusted odds ratio. COPD = chronic obstructive pulmonary disease. IMD = Index of Multiple Deprivation. TIA = transient ischaemic attack.

During the pandemic, 20.7% (*n* = 164 565) of adults living with T2D and 24.7% (*n* = 355 240) of adults living with hypertension gained weight rapidly (Figure 5).

Among adults with T2D, sociodemographic factors associated with rapid weight gain included the following: sex, the prevalence was highest among females (23.0%), while male sex reduced the likelihood (male versus female: aOR 0.78 [95% confidence interval [CI] = 0.77 to 0.79]); age, the prevalence was highest among those aged 18–29 years (30.4%), while older age reduced the likelihood (for example, 60–69 years versus 18–29 years: aOR 0.66 [95% CI = 0.61 to 0.71]); deprivation, prevalence was highest among adults living in the most deprived IMD quintile (22.0%), while living in less deprived areas reduced the likelihood (IMD 5 versus IMD 1: aOR 0.87 [95% CI = 0.85 to 0.89]); and ethnicity, a slightly higher proportion of Black than White adults gained weight rapidly (22.2% versus 21.3%), but Black adults had a lower likelihood of rapid weight gain in analyses adjusted for age, sex, and deprivation (Black versus White: aOR 0.95 [95% CI = 0.92 to 0.98]) (Figure 5 and Supplementary Table S4).

Adults with T2D and comorbid conditions had an increased likelihood of rapid weight gain, with mental health conditions associated with the greatest estimated effect (for example, depression: aOR 1.13 [95% CI = 1.12 to 1.15]). The prescribed diabetes treatment was also associated with likelihood of rapid weight gain: adults prescribed non-insulin treatment had a reduced likelihood compared with those who had no pharmacological treatment (aOR 0.68 [95% CI = 0.67 to 0.69]), while those prescribed insulin had an increased likelihood (aOR 1.16 [95% CI = 1.14 to 1.18]) (Figure 5).

These sociodemographic and clinical associations were maintained in a multivariate model mutually adjusted for age, sex, IMD, ethnicity, and diabetes medication (Supplementary Figure S2). Similar factors increased the adjusted odds of rapid weight gain among adults living with hypertension (Figure 5). But, consistent with findings in T2D, among adults with hypertension, those with comorbid T2D had lower likelihood of rapid weight gain compared with those without (aOR 0.74 [95% CI = 0.74 to 0.75]).

Analyses of pre-pandemic data were conducted to contextualise these findings. These analyses demonstrated that, before the onset of the pandemic, the prevalence of rapid weight gain was lower among adults with T2D (19.2%) and hypertension (21.8%); however, patterns of associations with the clinical and sociodemographic characteristics were similar (Supplementary Figure S3).

### Subgroup analyses

Trends seen in the whole population of adults living with T2D and/or hypertension were maintained in the analyses stratified by sex, ethnicity, and age (Supplementary Figures S4–S9).

## Discussion

### Summary

To the authors’ knowledge, this is the first large-scale analysis of weight trends among adults living in England with T2D and/or hypertension during the pandemic. Obesity was common among adults living with these conditions. However, people living with T2D tended to lose weight during the pandemic, while those living with hypertension maintained a stable weight. These overall trends disguised highly variable individual-level BMI trajectories; more than one-fifth of adults with T2D and almost one-quarter of those with hypertension gained weight rapidly (>0.5 kg/m^2^/year) during the pandemic. The characteristics associated with rapid weight gain were similar in both populations, with the highest likelihood among females, younger adults, those living in the most deprived areas, adults of White ethnicity, and those living with comorbid mental health conditions.

### Strength and limitations

The key strengths of this study are the quality, scale, and representativeness of the data used.^14^ In England, primary care hosts and records data from >90% of all patient consultations in the NHS.^15^ OpenSAFELY-TPP provides access to primary care records of roughly 40% of the population.^14^ The large sample size supports the generalisability of these findings and allowed patterns of weight gain in many sociodemographic and clinical strata to be investigated, which uncovered novel findings.

Although BMI data are not entered into the EHR following a predefined study protocol, routine EHRs provide BMI trajectory estimates comparable with those from population-based surveys.^16^ Adults with T2D are more likely to have their BMI recorded as this is part of the annual diabetes review.^17^ Restructuring of healthcare services during the pandemic may have introduced new bias.^9^^,^^18^ However, the present study reported that a high proportion of adults with T2D continued to have BMI recorded (Supplementary Table S1). The BMI data for adults living with hypertension were less complete and may be at greater risk of information bias (Supplementary Table S2).

The work has limitations. Increased BMI self-reporting during remote consultations may have introduced biases.^19^ The study was structured as an analysis of data from patients who were registered with a GP at the time of data extraction. This provided robust estimates of the current burden of obesity and rapid weight gain but may have introduced survival bias. A complete-case analysis was undertaken, which may preclude the generalisability of these findings to individuals with missing ethnicity or IMD data. Notably, a low proportion of adults had missing data in these fields.

### Comparison with existing literature

Data on weight trajectories among people living with hypertension are lacking, and novel evidence is presented that, on average, this population maintained a stable weight during the pandemic. The study’s finding that, on average, adults living with T2D in England lost weight during the pandemic has several possible disease-specific explanatory pathways. Diabetes and obesity were identified as independent risk factors for severe disease with COVID-19.^6^ Therefore, people with T2D may have made positive behavioural changes promoting weight loss, as seen in other European settings.^20^^,^^21^ The present study found that non-insulin treatment reduced the likelihood of rapid weight gain. Notably, prescription of novel diabetes drugs associated with weight loss (including sodium glucose co-transporter-2 inhibitors) increased in England during the pandemic (Supplementary Information S4). Alternatively, weight loss can be a sign of poorly controlled diabetes and may reflect worsening glycaemic control among adults with T2D. Further research is welcomed on investigating the impact of the pandemic on glycaemic control.

A high proportion of adults with T2D and/or hypertension gained weight rapidly, with the likelihood of rapid pandemic weight gain greatest among females, young adults, those living in more deprived areas, and those with mental health conditions. These patterns mirror those seen in the general population^9^ and provide further evidence of pandemic health inequalities, which have been described in relation to sex, ethnicity, wealth, employment, and education.^22^ The results of the present study support previous reports that young adults are at high risk of weight gain.^8^^,^^9^ The increased odds among females may reflect gender disparities in patterns of employment and caring responsibilities during the pandemic.^23^ Associations with deprivation are likely multifactorial in aetiology, including food poverty, reduced opportunity for physical activity, and an increased burden of physical and mental health conditions.^24^ The present study’s novel findings that comorbid mental health conditions increased odds of rapid weight gain among adults with T2D and/or hypertension may reflect associations between disordered eating, reduced physical activity, and poor mental health exacerbated by the pandemic.^25^^,^^26^

### Implications for research and practice

Clear characteristics associated with odds of rapid weight gain that could enable targeting of existing weight-management interventions have been identified.^10^ GPs are at the frontline of health care in the UK. They play a key role in supporting weight management through brief interventions and onward referral to weight-management programmes.^27^ The population subgroups that were at greatest risk of unhealthy patterns of weight gain during the pandemic and may consequently benefit the most from weight-management interventions have been highlighted. The findings — that comorbid mental health conditions are both prevalent among adults living with T2D and/or hypertension and are associated with increased odds of rapid weight gain — have highlighted the complex interplay between physical health, mental health, and weight. Future research and policy in this field must incorporate sociodemographic, physical, and mental health characteristics in prioritisation and implementation of interventions to ensure equity.

## Data

Access to the underlying identifiable and potentially re-identifiable pseudonymised electronic health record data is tightly governed by various legislative and regulatory frameworks, and restricted by best practice. The data in the NHS England OpenSAFELY COVID-19 service inside TPP are drawn from general practice data across England where TPP is the data processor (see Supplementary Information S5 for further details). All code for the full data management pipeline — from raw data to completed results for this analysis — and for the OpenSAFELY platform as a whole is available for review at: https://github.com/OpenSAFELY. The data management and analysis code for this article was led by Miriam Samuel and Robin Y Park and is available for scientific review and re-use under MIT open licence: https://github.com/opensafely/BMI-and-Metabolic-Markers.
